# Tri-valorization of methanol in a single bioreactor: co-production of enzyme, chemical, and single-cell protein using engineered *Pichia pastoris* (*Komagataella phaffii*)

**DOI:** 10.1186/s40643-026-01099-0

**Published:** 2026-07-13

**Authors:** Jiayu Fang, Shuxian Wang, Guoxia Liu, Yanping Zhang, Yin Li, Taicheng Zhu

**Affiliations:** 1 State Key Laboratory of Microbial Diversity and Innovative Utilization, Institute of Microbiology, Chinese Academy of Sciences, Beijing, 100101 China; 2 University of Chinese Academy of Sciences, Beijing, 100049 China

**Keywords:** *Pichia pastoris* (*Komagataella phaffii*), Methanol feedstock, Co-production strategy, Single-cell protein, Industrial enzymes, Erythritol

## Abstract

The economic viability of methanol-based biomanufacturing, particularly with green methanol as feedstock, is often limited by the low value of single-product processes. Here, we developed an integrated co-production strategy in the methylotrophic yeast *Pichia pastoris* (*Komagataella phaffii*) for the simultaneous conversion of methanol into three products: the sweetener erythritol, the industrial biocatalyst β-mannanase, and single-cell protein (SCP) biomass. This new strategy explores the inherent spatial and functional separation between the ER-Golgi secretory pathway for enzyme production and the cytosolic pathway for chemical synthesis, thereby reducing interference between the two pathways. The engineered co-production strain achieved β-mannanase and erythritol titers comparable to those of the corresponding β-mannanase- and erythritol-producing reference strains in both shake-flask and fed-batch fermentor cultures. Furthermore, transcriptomic analysis revealed distinct regulatory responses related to enzyme production and erythritol synthesis, supporting the limited cross-pathway interference between the two pathways. In addition, we showed that ultrafiltration enabled efficient downstream separation of the small-molecule erythritol from the secreted β-mannanase, with recovery efficiencies exceeding 89%. These results demonstrate a feasible strategy for methanol valorization into multiple value-added products and expand the potential of methylotrophic yeasts for integrated biomanufacturing.

**Graphical abstract:**

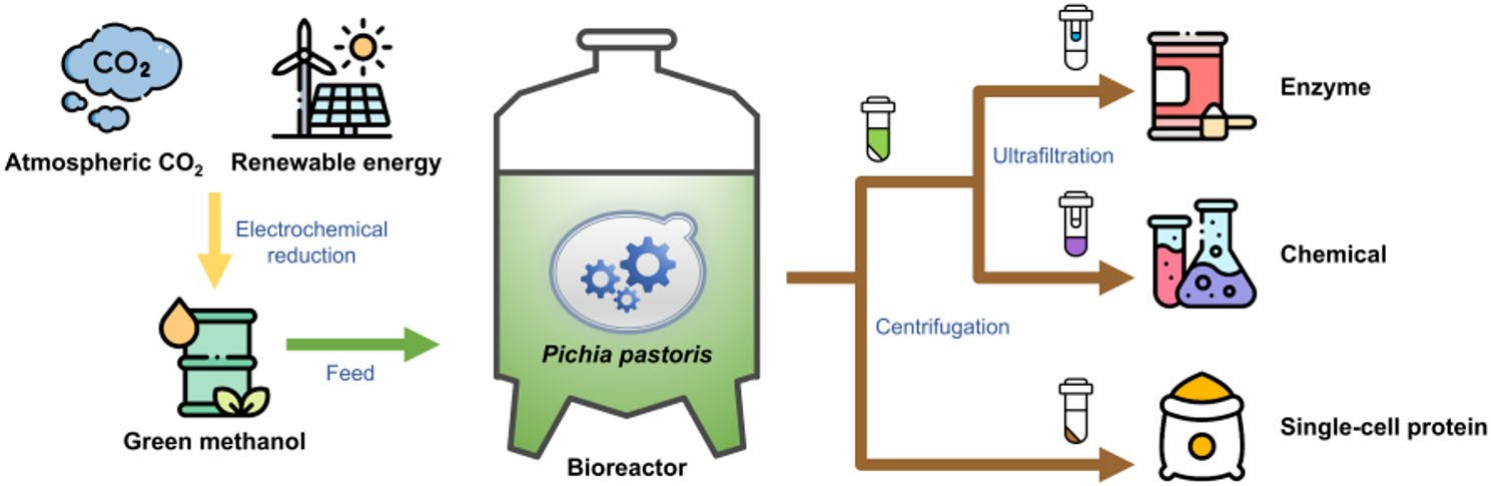

**Supplementary Information:**

The online version contains supplementary material available at 10.1186/s40643-026-01099-0.

## Introduction

The global demand for sustainable and environmentally friendly biomanufacturing processes has spurred interest in alternative carbon feedstocks (Orsi et al. 2023; Vogt and Weckhuysen 2024; Zhang et al. 2025). Among these, methanol has emerged as a promising candidate, particularly when produced sustainably as “green methanol” (Chen and Lan 2020; Jiang et al. 2021; Liu et al. 2023; Lv et al. 2023). Green methanol is derived from carbon dioxide (CO_2_) captured from the atmosphere or industrial emissions using renewable energy sources (Fasihi and Breyer 2024; Güleroglu and Yumurtaci 2025; Wang et al. 2023b). This process not only reduces greenhouse gas emissions but also provides a sustainable feedstock for a circular carbon economy, where waste CO_2_ is recycled into valuable products (Orsi et al. 2023; Wang et al. 2025b).

The methylotrophic yeast *Pichia pastoris* (syn. *Komagataella phaffii*) is exceptionally well-suited for methanol-based bioprocesses (Guo et al. 2023; Moraes et al. 2024; Vijayakumar and Venkataraman 2024; Zhou et al. 2025). In the 1970s, its potential for single-cell protein (SCP) production was first explored using fossil methanol to produce microbial proteins (Ritala et al. 2017). However, the commercialization of SCP from *P*. *pastoris* was hindered by rising oil and gas costs (Groenewald et al. 2014; Johnson 2013). In recent years, the increasing demand for artificial foods and the focus on reducing CO_2_ emissions have generated renewed interest in SCP production from C1 feedstocks (Gao et al. 2023; Meng et al. 2023). Despite the environmental advantages, SCP production from methanol—especially green methanol—faces significant economic challenges due to high feedstock costs. In contrast, production from gas fermentation can be more cost-effective, especially using waste gases like methane and CO (Li et al. 2023; Vlaeminck et al. 2023).

In the 1990s, *P*. *pastoris* gained recognition as an efficient protein expression system, eventually becoming one of the most successful platforms in industrial biotechnology (Ergün et al. 2022). It is now widely used for large-scale enzyme production, with products like phytases, proteases, mannanases, and lipases produced in quantities ranging from several tons to hundreds of tons (Ergün et al. 2021; Pan et al. 2022; Yang and Zhang 2018; Zhu et al. 2019). This large-scale enzyme production offers a pathway to valorize methanol, as it can be combined with SCP production to improve economic viability (Yan et al. 2018). However, the bulk enzyme market is highly competitive, and price reductions—some enzymes now costing less than $1 per kilogram—pose challenges for profitability when methanol is used as a feedstock.

Beyond protein and enzyme production, *P*. *pastoris* has also been developed as a microbial cell factory for chemical production (Guo et al. 2023; Wang et al. 2023a, 2025b; Zha et al. 2023). Advances in metabolic engineering have enabled the yeast to produce chemicals at titers exceeding 1 g/L, with some studies reporting production levels in the tens of grams per liter (Wang et al. 2025b; Wu et al. 2023; Yang et al. 2024). The co-production of chemicals with SCP represents a new approach to valorizing C1 feedstocks, similar to co-production in gas fermentation processes (Vlaeminck et al. 2023). However, the relatively low yields and titers of chemicals produced by *P*. *pastoris* still limit the commercial feasibility of co-producing chemicals and SCP on a large scale (Guo et al. 2021; Wang et al. 2025b).

As such, each of these strategies—SCP, enzyme, or chemical production—faces its own economic limitations when pursued individually. This highlights a clear need for a more integrated approach to improve the overall process value. To address this challenge, we propose a tri-valorization strategy: the simultaneous production of SCP (biomass), an industrial enzyme, and a bulk chemical within a single chassis. The rationale for this approach lies in the limited spatial interference between these metabolic tasks. Enzyme secretion relies on the endoplasmic reticulum (ER) and Golgi apparatus, while chemical synthesis typically occurs in the cytosol (Huttanus and Feng 2017). This subcellular compartmentalization suggests that these processes can operate in parallel with limited direct interference. Furthermore, the significant size difference between proteins and small-molecule chemicals allows for their straightforward separation using simple techniques like ultrafiltration (Kammakakam and Lai 2023).

While this co-production concept is promising, it also raises concerns about whether competition for cellular resources, such as energy and metabolic precursors, might compromise overall productivity. In this study, we address these questions by establishing a proof-of-concept system in *P*. *pastoris* for the co-production of an industrially relevant enzyme, β-mannanase, and a value-added chemical, erythritol. Erythritol is an important bulk sweetener with potential as a C4 platform chemical, whereas β-mannanase is a widely used industrial enzyme with relevance to biomass and food/feed processing (Wang et al. 2025a; Zhu et al. 2011). By integrating the cytosolic synthesis pathway for erythritol with the secretory pathway for β-mannanase, we demonstrate the feasibility of a tri-valorization strategy that converts methanol into three distinct product streams. This work provides a framework for enhancing the economic viability of methanol-based biomanufacturing and for developing more versatile methylotrophic cell factories.

## Materials and methods

### Construction of co-production strain

Two previously engineered *P*. *pastoris* strains served as the basis for this study. The first strain, originally named GS-4sman (Zhu et al. 2011), produced β-mannanase extracellularly and is referred to here as GS-M. The strain contains four tandemly integrated copies of the β-mannanase gene. The second strain, designated GS-A, was previously reported as GS-2AY (Wang et al. 2025a) and was engineered for erythritol production. The GS-A strain was generated by transforming wild-type *P*. *pastoris* with the plasmid pPICHhis-2AY, which contains two copies each of the erythrose-4-phosphate phosphatase (YidA) and erythrose reductase (ALR) genes. To construct the co-production strain (GS-AM), the GS-M was transformed with the linearized plasmid pPICHhis-2AY, which is identical to the one used to create the parental GS-A strain.

### Erythritol and β-mannanase production by shake flask culture

Unless otherwise noted, all strains were first precultured in YPD medium (10 g/L yeast extract, 20 g/L peptone, 20 g/L glucose) for 48 h and transferred into 250 mL baffled shake flasks containing 25 mL BMMY medium (8.7 g/L potassium phosphate, 13.4 g/L yeast nitrogen base, 20 g/L peptone, 10 g/L yeast extract, 0.4 mg/L biotin, pH 6.0) at 30 °C. Erythritol and β-mannanase production was initiated by adding 10 g/L methanol, with additional 10 g/L methanol added every 12 h. Three independent biological replicates were performed.

### Erythritol and β-mannanase production by fed-batch culture in fermentor

Fed-batch fermentations were performed in a 1-L fermentors (Infors, Switzerland) containing 0.8 L BMGY medium (8.7 g/L potassium phosphate, 13.4 g/L yeast nitrogen base, 20 g/L peptone, 10 g/L yeast extract, 0.4 mg/L biotin, 40 g/L glycerol, pH 6.0), supplemented with 3.2 mL PTM1 trace salts (6 g/L CuSO_4_⋅5H_2_O, 0.09 g/L KI, 3 g/L MnSO_4_⋅H_2_O, 0.02 g/L H_3_BO_3_, 0.2 g/L Na_2_MoO_4_⋅2H_2_O, 0.5 g/L CoCl_2_, 20 g/L ZnCl_2_, 65 g/L FeSO_4_⋅7H_2_O, 0.2 g/L biotin, 5.0 mL/L H_2_SO_4_).

The temperature was maintained at 30 °C, pH was controlled at 6.0 by adding NH_3_·H_2_O (28%, v/v), and the air flow rate was set to 2 L/min. Strains were inoculated after preculture in YPD medium. The fermentation began with the glycerol phase, which continued until glycerol was depleted. Methanol induction was then initiated by adding 3 g/L methanol. During the methanol-feeding phase, the residual methanol concentration was maintained at 3 g/L using an automatic methanol control system (FC2002, East China University of Science and Technology, Shanghai, China) that fed methanol containing 1.2% PTM1 trace salts.

### Analytic methods

The erythritol titers were measured by HPLC analysis using an Aminex HPX-87 H column (Bio-Rad) with 8 mM H_2_SO_4_ as the mobile phase. The flow rate was set to 0.6 mL/min, and the column temperature was maintained at 55 °C. Erythritol concentrations were detected using a refractive index (RI) detector.

β-mannanase activity was measured as described by Zhu et al. (Zhu et al. 2011). The reaction system consisted of 190 µL of 0.05 M glycine-NaOH buffer (pH 9.0) containing 0.5% locust bean gum, to which 10 µL of suitably diluted culture supernatant was added. The mixture was incubated at 70 °C for 5 min. Reducing sugar concentration was then determined using the 3,5-dinitrosalicylic acid (DNS) method. One unit of β-mannanase activity was defined as the amount of enzyme that liberated 1 µmol of reducing sugar per minute using locust bean gum as the substrate.

### Separation of erythritol and β-mannanase by ultrafiltration

The fermentation broth was first centrifuged at 13,000×*g* for 1 min to remove cellular debris. The supernatant was then subjected to ultrafiltration using Amicon Ultra-0.5 centrifugal filters (Millipore) with two different molecular weight cut-off (MWCO) membranes (3 kDa and 10 kDa) to separate erythritol and β-mannanase. The permeate was enriched with erythritol, while the retentate contained β-mannanase. The separation efficiency for each product was calculated by measuring the product titers in both the permeate (erythritol) and retentate (β-mannanase) fractions.

### Transcriptome analysis

mRNA sequencing and raw data analysis were performed by Novogene Co., Ltd (Beijing, China).

### Analysis of gene expression by quantitative real-time PCR (RT-qPCR)

Total RNA was extracted using the Yeast RNAiso Kit (TaKaRa, Japan). cDNA was synthesized using the SynScript^®^Ⅲ RT SuperMix for qPCR (Tsingke, China). The RT-qPCR was performed using the FastFire qPCR PreMix (TIANGEN, China) on a LightCycler^®^ 96 Instrument (Roche, Switzerland). The *ACT1* gene, which encodes actin, served as an internal control. For target amplification, the following primers were used: ALR-F (5’-catcgagtctggctaccgtc-3’) and ALR-R (5’-gtccctcttcagggttgtcg-3’) for the *ALR* gene, yidA-F (5’-atcactggccgacgtgttag-3’) and yidA-R (5’-ttatccatcgccacaccgac-3’) for the *yidA* gene, manA-F (5’-aacctccaaaccatccaccg-3’) and manA-R (5’-acccatcacgttgcttccat-3’) for the *manA* gene, and actin-F (5’-ggagcaggaaactgaggagg-3’) and actin-R (5’-ttcgcctgtgtatggctctg-3’) for the *ACT1* gene. Relative expression levels were calculated using the 2^−ΔΔCt^ method.

## Results

### Engineering *Pichia pastoris* for co-production of erythritol and β-mannanase

We previously reported high-level expression of an alkaline β-mannanase in *P*. *pastoris*, an enzyme with important industrial applications in the pulp and paper industry (Zhu et al. 2011). More recently, we demonstrated the successful construction of a *P*. *pastoris*-based chemical cell factory for erythritol production—erythritol being a bulk sweetener and a potential platform chemical for synthesizing various C4 derivatives (e.g., butanediol, butanetriol) via chemical conversion (Wang et al. 2025a).

In this study, the GS-4sman strain, carrying four tandem copies of the β-mannanase gene, was used as the reference strain for enzyme production and designated GS-M. The erythritol-producing control strain (GS-A) was generated by transforming the wild-type strain with pPICHhis-2AY, which carries two copies each of erythrose-4-phosphate phosphatase (YidA) gene and erythrose reductase (ALR) gene. For co-production, the pPICHhis-2AY plasmid was introduced into GS-M, yielding the co-production strain GS-AM.

**Fig. 1:**
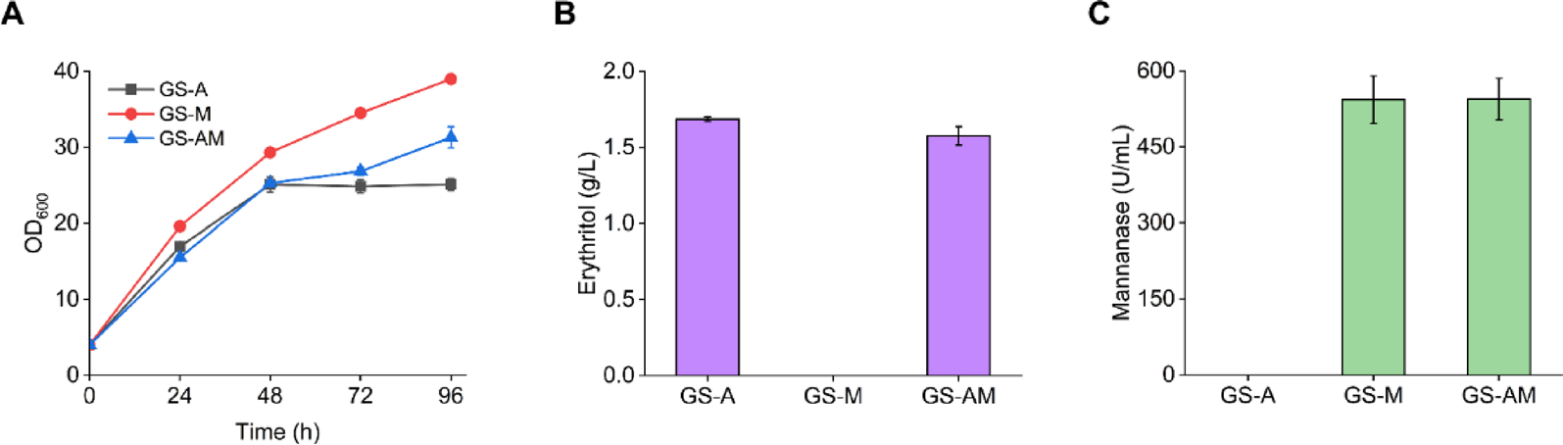
Co-production of erythritol and β-mannanase by engineered *Pichia pastoris*. **A** Growth profile of erythritol-producing strain GS-A, β-mannanase-producing strain GS-M and co-production strain GS-AM. **B** Erythritol production of GS-A and GS-AM. **C** β-mannanase production of GS-M and GS-AM. Three independent biological replicates were performed

All three strains (GS-M, GS-A, and GS-AM) were evaluated in shake-flask cultures with methanol feeding every 12 h. Results showed that there was no significant difference in β‑mannanase production between GS‑AM and GS‑M (Fig. 1C). However, growth profiling revealed that GS-AM grew markedly slower than GS-M, suggesting that diversion of carbon flux to erythritol synthesis reduced resources available for biomass formation—consistent with our previous observations (Fig. 1A). As a result, β-mannanase production per OD_600_ was higher in GS-AM than in GS-M.

Erythritol production in GS-AM was also similar to that in GS-A (Fig. 1B). However, because GS-AM exhibited a slightly higher growth rate than GS-A, erythritol yield per OD_600_ was marginally lower, indicating that the erythritol production capacity of the co-production strain was slightly reduced compared with the erythritol-only strain.

### Effect of temperature and dissolved oxygen on the co-production of erythritol and β-mannanase

Temperature and dissolved oxygen (DO) are generally considered important process parameters affecting fermentation performance for enzyme production (De Brabander et al. 2023), but their influence on chemical production—and particularly on co-production—has been less investigated. In this work, we examined the effects of these two parameters on the three strains.

**Fig. 2:**
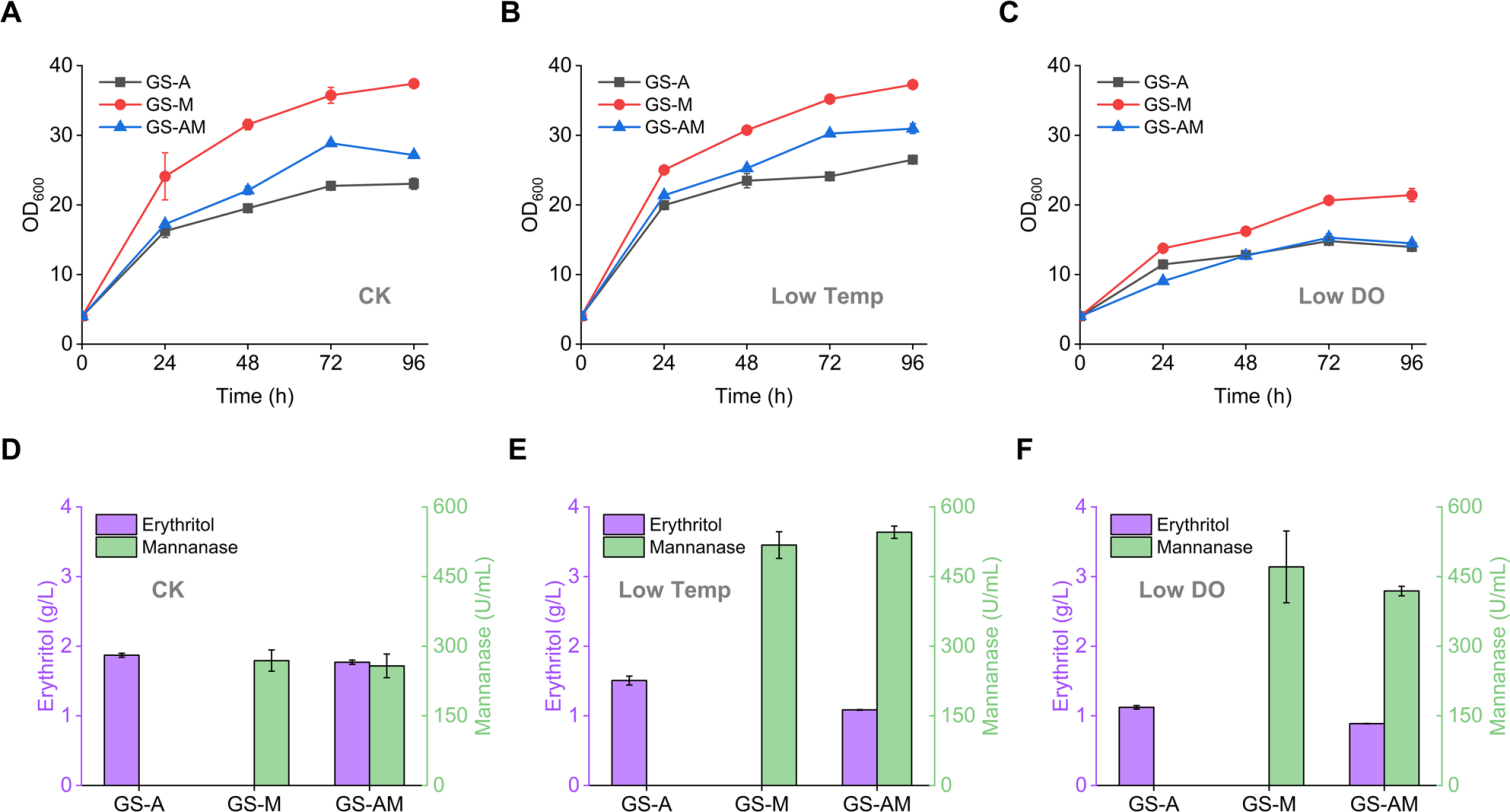
Performance of co-production strain GS-AM in low temperature or low dissolved oxygen conditions. **A–C** Growth profile of erythritol-producing strain GS-A, β-mannanase-producing strain GS-M and co-production strain GS-AM cultured in different culture conditions. **D–F** Comparisons of erythritol or β-mannanase production between single-production strain GS-A/GS-M and co-production strain GS-AM in different culture conditions. CK: 250 mL baffled shaking flasks with a 10% working volume at 30 °C; Low Temp: 250 mL baffled shaking flasks with a 10% working volume at 26 °C; Low DO: 100 mL baffled shaking flasks with a 20% working volume at 30 °C. Three independent biological replicates were performed

For the β-mannanase-producing strain GS-M, lowering the temperature to 26 °C and reducing DO by using a 20% working volume resulted in β-mannanase activity increases of 92.6% and 75.1%, respectively (Fig. 2D and F). In contrast, for erythritol production in GS-A, both significantly decreased erythritol titers by 19.5% and 40.0%, respectively (Fig. 2D and F). For the co-production strain GS-AM, the effects of temperature and DO on β-mannanase and erythritol production were consistent with those observed in GS-M and GS-A, respectively (Fig. 2D and F).

Regarding biomass accumulation, reducing DO caused a marked decrease in biomass across all three strains, whereas lowering the temperature to 26 °C led to only a minor decrease compared with 30 °C (Fig. 2A and C). This suggests that both lower temperature and reduced DO likely slow the methanol metabolic rate, thereby reducing cell growth rate. However, lower temperature often leads to a higher biomass yield on methanol (Sirén et al. 2006; Zhu et al. 2011), which can compensate for the reduced metabolic rate and explains the only slightly reduced biomass levels observed. The slower methanol metabolism under these conditions also decreases the carbon flux available for product synthesis, accounting for the drop in erythritol titer. For β-mannanase, where secretion rather than synthesis is often the limiting step (Zhu et al. 2011), a reduced production load may alleviate folding and secretion stress, enabling more efficient enzyme export and resulting in higher extracellular activity.

### Co-production of erythritol and β-mannanase in fermentor culture

Given that the co-production strain demonstrated comparable enzyme and chemical production capabilities to the corresponding single-production strains in shake-flask culture, its scalability was further assessed in a 1-L fermentor with the single-production strains as controls. During the glycerol batch phase (data not shown), all three strains exhibited similar performance, reaching an OD_600_ of approximately 40 before transitioning to the methanol phase for cell growth and product synthesis.

**Fig. 3:**
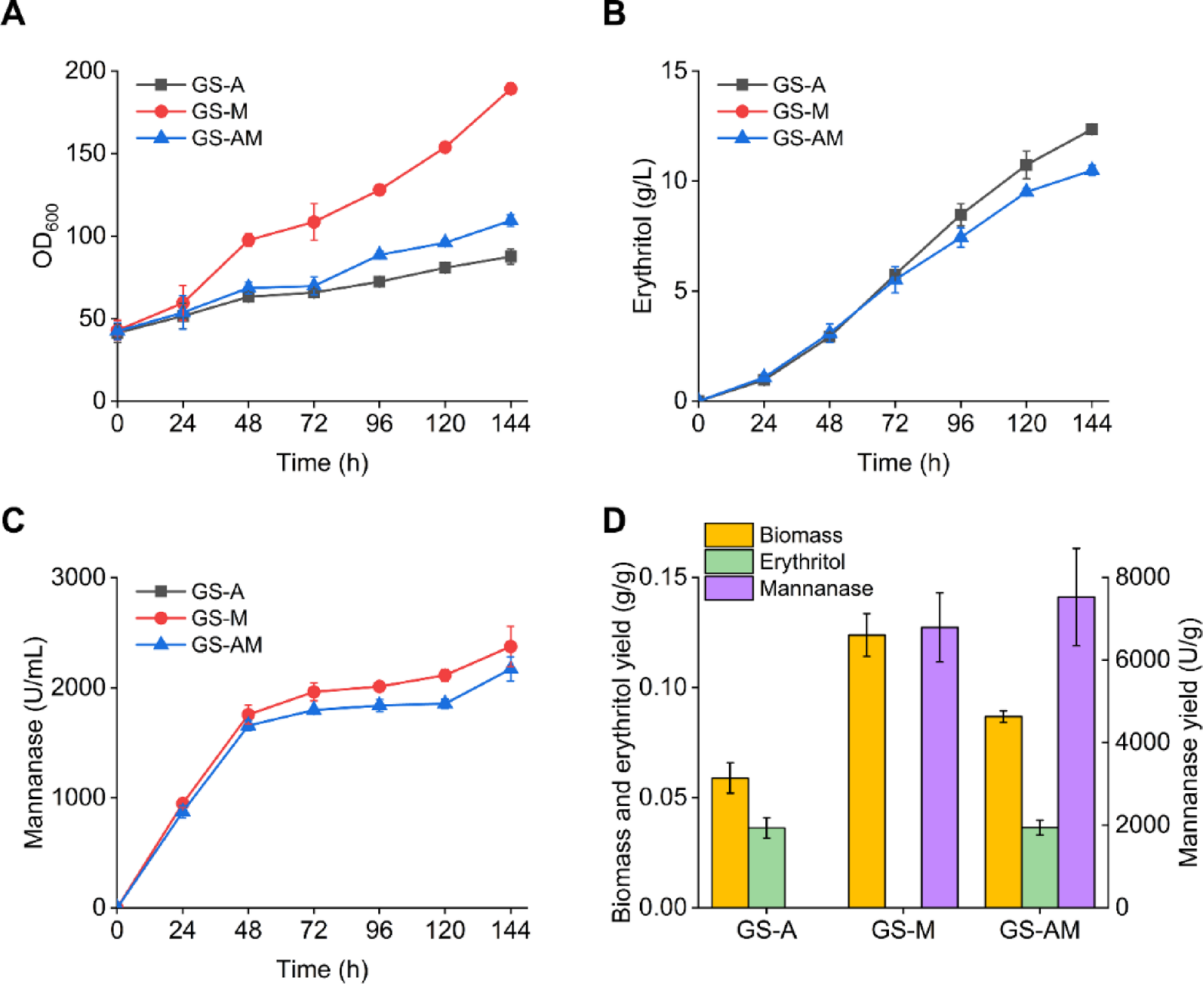
Co-production of erythritol and β-mannanase by GS-AM strain in 1-L fermentor. **A** Growth profile of erythritol-producing strain GS-A, β-mannanase-producing strain GS-M and co-production strain GS-AM. **B** Erythritol production of GS-A and GS-AM strain. **C** β-mannanase production of GS-M and GS-AM strain. **D** Product yields of GS-A, GS-M and GS-AM strains. Biomass yield was defined as the grams of dry cell weight produced per gram of methanol consumed; erythritol yield was defined as the grams of erythritol produced per gram of methanol consumed; β-mannanase yield was defined as the units of mannanase activity produced per gram of methanol consumed. All experiments were performed in biological duplicate

In the methanol phase, growth patterns of all strains mirrored those observed in shake-flask culture (Figs. 1A and 3A). The GS-AM strain accumulated less biomass than GS-M but more than GS-A (Fig. 3A). For product formation, GS-AM showed slightly reduced titers compared to the single-production strains (Fig. 3B and C). After 144 h of methanol induction, erythritol and β-mannanase titers in GS-AM reached 84.9% and 91.4% of those obtained from GS-A and GS-M, respectively.

Biomass and product yields on methanol were also calculated for all three strains. Compared with GS-A, GS-AM exhibited a biomass yield increase of 47.4%, while the erythritol yield on methanol remained nearly unchanged. Compared with GS-M, GS-AM showed a biomass yield reduction of 29.9%, while β-mannanase yield remained at the same level. These results indicate that co-production in *P*. *pastoris* scales effectively to fermentor conditions with only minor trade-offs, supporting efficient carbon allocation to both erythritol and β-mannanase without substantial pathway interference.

In addition, the crude protein content of the biomass differed among the three strains and was inversely related to their biomass yield (Fig. 3D, S1). The fast-growing GS-M, which directed the most carbon into biomass, showed the lowest protein content (0.521 g/g DCW), whereas the slow-growing GS-A, in which carbon was largely diverted to erythritol, accumulated the most nitrogen-rich biomass (0.587 g/g DCW). The co-production strain GS-AM displayed an intermediate value (0.567 g/g DCW), confirming that the biomass remains a valuable protein co-product even under the co-production scenario.

### Physiological investigation of the co-production strain with transcriptional analysis

**Fig. 4:**
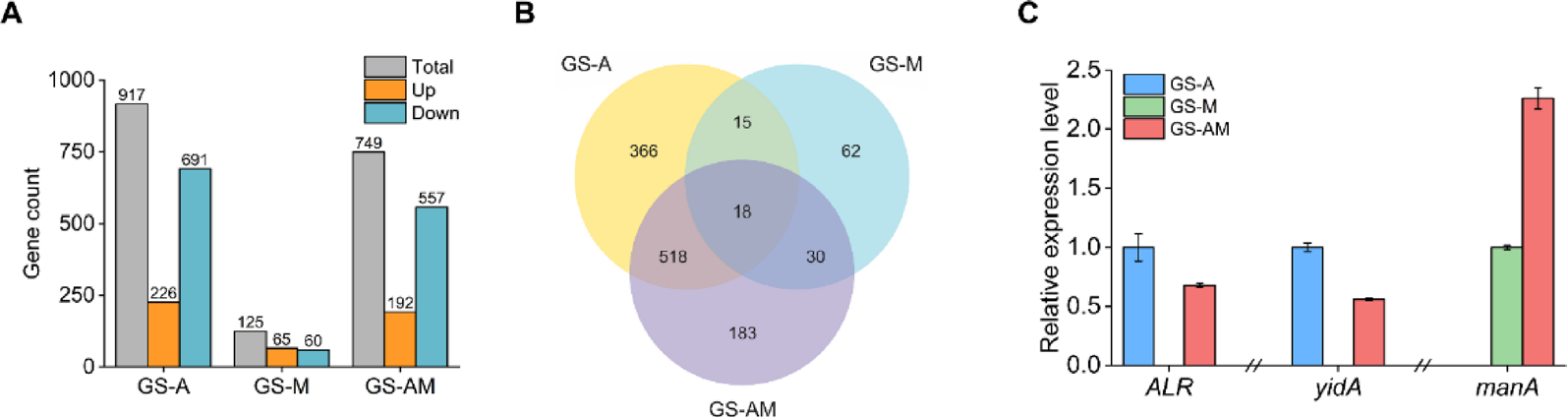
Transcriptome analysis of co-production strain and single-production strains. **A** Statistical chart of differentially expressed genes in single-production strains GS-A, GS-M, and co-production strain GS-AM, with wild-type GS115 strain as the control. **B** Venn diagram of the total number of differentially expressed genes in three comparison groups. **C** Relative expression levels of erythritol synthesis genes *ALR*, *yidA* and β-mannanase synthesis gene *manA* in GS-A, GS-M and GS-AM strains. Relative expression levels of genes are shown after normalization to the corresponding single-production strain

To investigate the effects of co-production on the physiology of the co-production strain and compare it with single-production strains at the molecular level, we performed qPCR and transcriptome analyses of GS-AM alongside GS-A and GS-M. Cells were collected at 36 h of induction in shake flasks for analysis. First, qPCR quantification of heterologous genes revealed that the erythritol synthesis genes (*ALR* and *yidA*) in GS-AM were down-regulated compared to GS-A, while the β-mannanase synthesis gene *manA* was up-regulated relative to GS-M (Fig. 4C). These changes explain the observed decrease in erythritol production per OD_600_ and the increase in β-mannanase production per OD_600_ observed previously in shake flask culture.

Next, to reveal the physiological changes in single or co-production strains, transcriptome analysis was performed using the WT strain as a control. Results showed that GS-A regulated 917 genes, GS-M regulated 125 genes, and GS-AM regulated 749 genes (Fig. 4A, S2), suggesting that erythritol production caused substantially greater physiological perturbation than β-mannanase production, which is consistent with the reduced growth of GS-A. GO enrichment analysis revealed that, compared with the WT strain, GS-M up-regulated ribosome biogenesis (GO:0042254), protein folding in the ER (GO:0034975), thiamine-diphosphate biosynthesis (GO:0009229), and lipid metabolism (GO:0006629) to expand translation and secretion capacity for β-mannanase expression, while down-regulating amino acid catabolism and oxidative pathways to conserve nitrogen and NAD(P)H (Fig. S3). In contrast, GS-A strongly induced glycolysis (GO:0006096), oxidation-reduction processes (GO:0055114), and thiamine-related biosynthesis (GO:0009228/0009229), while suppressing ribosome biogenesis, mitochondrial translation, and DNA replication—reflecting a trade-off between growth and erythritol synthesis (Fig. S3).

Moreover, only 33 differentially expressed genes (DEGs) were shared between GS-A and GS-M (Fig. 4B), suggesting that the biosynthesis of erythritol and β-mannanase exhibit limited cross-pathway interference, likely operating through distinct regulatory mechanisms, as assumed. Nevertheless, a comparison of the GS-AM strain with either GS-A or GS-M (Fig. 4B) revealed that the DEG set of GS-AM is not a simple combination of the changes observed in GS-A and GS-M. GS-AM exhibited 183 independent DEGs that were absent from either single-production background. GO enrichment analysis showed that the co-production strain exclusively up-regulates phospholipid translocation (GO:0045332), cellular response to phosphate starvation (GO:0016036), and calcineurin-dependent stress signaling (e.g., GO:0071363), while down-regulating trans-sulfuration (GO:0006545), ribosome biogenesis (GO:0042254), and the G2/M cell-cycle transition (GO:0000086) (Fig. S3). These results suggested that the co-production strain, GS-AM, adjusts its metabolism to optimize membrane remodeling, stress response, and nutrient sensing, potentially balancing the production of both erythritol and β-mannanase. This regulatory shift may be necessary to handle the dual demands of erythritol synthesis and β-mannanase production while maintaining cellular homeostasis under the stress of co-production.

### Separation of erythritol and β-mannanase from fermentation broth

**Fig. 5:**
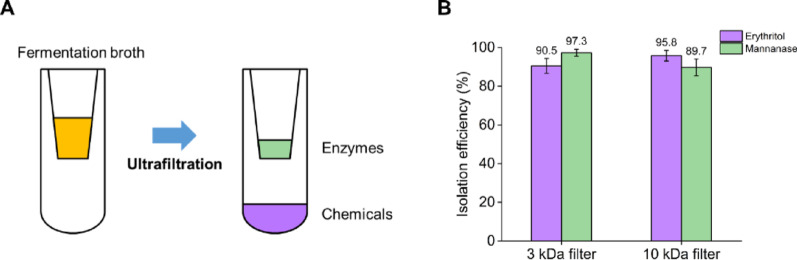
Separation of erythritol and β-mannanase from fermentation broth by ultrafiltration. **A** Diagram of the separation of erythritol and β-mannanase by ultrafiltration. The permeate was enriched with erythritol and the retentate was enriched with β-mannanase. **B** Isolation efficiency of erythritol and β-mannanase using ultrafiltration membranes with different MWCOs. The isolation efficiency was defined as the ratio of erythritol content or mannanase activity in the permeate/retentate relative to that in the fermentation broth

We hypothesized that the separation of erythritol and β-mannanase could be efficiently achieved by ultrafiltration due to the significant difference in molecular weight between the chemicals and proteins. To verify this concept, we tested two different ultrafiltration membrane sizes (3 kDa and 10 kDa) for the separation of erythritol and β-mannanase. The fermentation broth was collected by centrifugation and then subjected to ultrafiltration, resulting in a permeate enriched with erythritol and a retentate containing β-mannanase (Fig. 5A). The corresponding product titers in both fractions were measured to calculate the separation efficiency (Fig. 5B, S4).

The ultrafiltration method demonstrated high recovery rates (> 89%) for both erythritol and β-mannanase separation from the fermentation broth. Notably, membranes with a lower MWCO showed better recovery for the macromolecular enzyme (β-mannanase), while membranes with a higher MWCO were more suitable for the small-molecular chemical (erythritol). Therefore, to maximize total product revenue, the optimal selection of ultrafiltration membranes with appropriate MWCO should be systematically determined, taking into account the specific enzyme-chemical production combination and their corresponding market prices.

## Discussion

This study establishes a proof-of-concept tri-valorization strategy in *Pichia pastoris* for the simultaneous production of single-cell protein (SCP) biomass, β-mannanase, and erythritol from methanol. While co-production of enzymes and chemicals in microbial fermentation has been reported previously, all prior strategies have employed sugar- (Park et al. 2023; Shamala et al. 2012) or oil-based (Marsudi et al. 2008) carbon substrates, and the concept has never been extended to methanol as the sole carbon and energy source. To date, methanol-based bioconversion in this host has focused almost exclusively on single-product objectives—either recombinant enzyme expression or small-molecule biosynthesis. By integrating three distinct value streams within a single methanol-fed fermentation, this study provides both a conceptual framework and a practical route for overcoming the economic constraints that currently limit methanol-based biomanufacturing.

This co-production strategy was based on the central hypothesis that the limited spatial interference between the secretory pathway (for enzymes) and cytosolic pathways (for chemicals) would enable simultaneous production with minimal interference. Our results, using β-mannanase and erythritol as model products, largely support this hypothesis. In shake flask cultures, the co-production strain (GS-AM) achieved product titers comparable to those of the dedicated single-production strains. Transcriptome analysis further corroborated the hypothesis, revealing that the metabolic perturbations caused by erythritol synthesis (GS-A) and β-mannanase secretion (GS-M) were highly distinct. Notably, only 33 differentially expressed genes (DEGs) were shared between the two strains, and Gene Ontology (GO) enrichment analysis showed that the DEGs in these strains were associated with largely different biological processes.

Nevertheless, co-production is not a simple additive process to single-product production. qPCR analysis revealed that the erythritol synthesis genes (*ALR* and *yidA*) in GS-AM were down-regulated compared to the erythritol-only strain (GS-A), while the β-mannanase synthesis gene (*manA*) was up-regulated relative to the β-mannanase-only strain (GS-M). This corresponds with the observed decrease in erythritol production per OD_600_ and the increase in β-mannanase production per OD_600_ in the co-production strain. These changes may be due to titration effects caused by the multiple copies of the AOX1 promoter in the co-production strain (Cámara et al. 2017). Because the cellular pool of methanol-responsive transcriptional activators that drive the AOX1 promoter is finite, adding the two *ALR* and two *yidA* cassettes on top of the four β-mannanase copies already present in GS-M dilutes—or titrates—these limiting activators across a larger number of promoter copies, attenuating the average transcriptional output per cassette. These results suggest that the use of different promoters for each product may be necessary to further mitigate the interference between the two processes.

This co-production concept was further demonstrated by scaling up to fed-batch culture in a fermentor. The titers of erythritol and β-mannanase in the co-production strain remained high, reaching 84.9% and 91.4% of their respective single-producer counterparts. More importantly, due to reduced methanol utilization in the co-production strain (data not shown), the yields of erythritol and β-mannanase remained the same or even higher than in their respective single-product strains. In terms of biomass (SCP) yield, the co-production strain exhibited a yield similar to the erythritol-producing strain but was 29.9% lower than the β-mannanase-producing strain. Thus, compared to the sole enzyme production mode—already at industrial-scale production—the introduction of chemical synthesis does not negatively impact enzyme yield. The co-production process benefits from the additional production of value-added chemicals while incurring only a moderate reduction in SCP production. To examine how methanol-derived carbon was distributed among the products, we constructed a feedstock carbon balance based on the per-methanol product yields and the carbon mass fraction of each product (Fig. S5). Carbon yields toward erythritol and β-mannanase were essentially preserved in GS-AM (~ 3.8% and ~ 0.4% of methanol carbon, respectively), whereas the main redistribution occurred in biomass-associated carbon (15.8% in GS-M versus 11.1% in GS-AM). Consistent with this, intracellular NAD(P)H levels were comparable across the three strains (Fig. S6), indicating no measurable change in cofactor status. Together, these data show that adding chemical synthesis to the enzyme- and SCP-producing chassis redistributes carbon mainly at the level of biomass accumulation, rather than diverting it from the target products or imposing a detectable cofactor burden.

Conversely, compared with sole chemical production—which often struggles to break even at present—co-production adds an enzyme product at essentially no extra carbon cost. Lab-scale ultrafiltration experiments demonstrated that the enzyme can be efficiently recovered, indicating that co-production can substantially enhance process valorization, particularly for chemical production in methylotrophic yeast. Nevertheless, the present separation was performed at the laboratory scale, and industrial translation will require addressing the challenges of large-scale membrane operation—concentration polarization and fouling, periodic cleaning and membrane replacement, and selection of an appropriate molecular-weight cut-off for each enzyme-chemical pair. Even so, ultrafiltration is a mature and widely deployed unit operation in industrial bioprocessing, supporting the feasibility of the proposed strategy at scale.

To substantiate the economic argument, we performed a preliminary feedstock-based estimation of the product value generated per kilogram methanol for the three strains (Table S1). Based on the product titers obtained in the 1-L fed-batch fermentations and their market prices, the tri-valorization strain (GS-AM) generated approximately 0.69 USD per kg methanol, versus 0.58 USD for the β-mannanase strain (GS-M) and only 0.12 USD for the erythritol strain (GS-A). Using a representative methanol reference price (~ 0.286 USD/kg, mid-2025), GS-AM reached a feedstock-to-product value ratio of ~ 2.4-fold, 19.1% higher than GS-M (~ 2.0-fold) and far above GS-A, whose product value alone fails to cover the methanol cost. These numbers support the central argument that integrating an industrial enzyme into a chemical- and SCP-producing chassis is an effective way to push a methanol-based bioprocess across the economic break-even line.

It should be noted that this estimation is intended as a demonstration of the concept rather than a complete techno-economic analysis. Only the raw-material cost of methanol is considered, while utilities, labour, equipment depreciation, and downstream separation are not included. Moreover, erythritol is an illustrative rather than optimal target, as its current titer remains far from commercially competitive for a bulk chemical. Nevertheless, the underlying logic—coupling a high-value enzyme with a chemical that shares precursors of the central methanol assimilation pathway—can be readily extended to other products. For instance, aromatic compounds derived from the same erythrose-4-phosphate (E4P) node, such as dopamine (Fan et al. 2025), have reached 12.2 g/L in *P*. *pastoris* at unit prices roughly an order of magnitude above erythritol; co-producing such chemicals with an industrial enzyme on the same methanol-driven chassis should further improve the commercial potential of methanol-based biomanufacturing.

Overall, using β-mannanase and erythritol as model products, this work demonstrates that methanol can be simultaneously converted by engineered *P*. *pastoris* into SCP biomass, a secreted enzyme, and a small-molecule chemical with limited cross-pathway interference. Three features establish a practical framework for integrated methanol-based biomanufacturing: robust co-production at the fermentor scale, transcriptome-supported evidence for limited cross-pathway interference, and straightforward downstream separation of chemically distinct products. Together, these advances expand the potential of methylotrophic yeasts as versatile platforms for the co-production of multiple value-added products.

## Supplementary Information

Below is the link to the electronic supplementary material.

Supplementary Material 1

## Data Availability

Data are available from the corresponding author upon reasonable request.
