# Supplementary material for: Tri-valorization of methanol in a single bioreactor: co-production of enzyme, chemical, and single-cell protein using engineered Pichia pastoris (Komagataella phaffii)
Source: Bioresour Bioprocess. 2026 Jul 13;13(1):102. doi: 10.1186/s40643-026-01099-0 (PMC13365088; doi:10.1186/s40643-026-01099-0)
Supplement: Supplementary file 1 — Supplementary Material 1 [file 40643_2026_1099_MOESM1_ESM.docx]

Supporting information for:

**Tri-Valorization of Methanol in a Single Bioreactor: Co-Production of Enzyme, Chemical, and Single-Cell Protein Using Engineered *Pichia pastoris* (*Komagataella phaffii*)**

Jiayu Fang^1,2^, Shuxian Wang^1,2^, Guoxia Liu^1^, Yanping Zhang^1*^, Yin Li^1^, Taicheng Zhu^1*^

^1^ State Key Laboratory of Microbial Diversity and Innovative Utilization, Institute of Microbiology, Chinese Academy of Sciences, Beijing 100101, China

^2^ University of Chinese Academy of Sciences, Beijing 100049, China

* Corresponding author.

E-mail address: zhutc@im.ac.cn (T. Zhu); zhangyp@im.ac.cn (Y. Zhang)

**Figure S1.** Crude protein content of the biomass from the erythritol-producing (GS-A), β-mannanase-producing (GS-M) and co-production (GS-AM) strain.

**Figure S2.** Volcano plots of differentially expressed genes in GS-A, GS-M and GS-AM strain.

**Figure S3.** GO enrichment analysis of differentially expressed genes in GS-A, GS-M and GS-AM strain.

**Figure S4.** Erythritol concentration and β-mannanase activity before and after ultrafiltration.

**Figure S5.** Feedstock carbon balance of the single-production (GS-A, GS-M) and co-production (GS-AM) strain.

**Figure S6.** Determination of intracellular NAD(P)H levels in GS-A, GS-M and GS-AM strain.

**Table S1.** Preliminary feedstock-based estimation of the product value per kilogram methanol for the tri-valorization strain (GS-AM), β-mannanase-producing strain (GS-M) and erythritol-producing strain (GS-A).


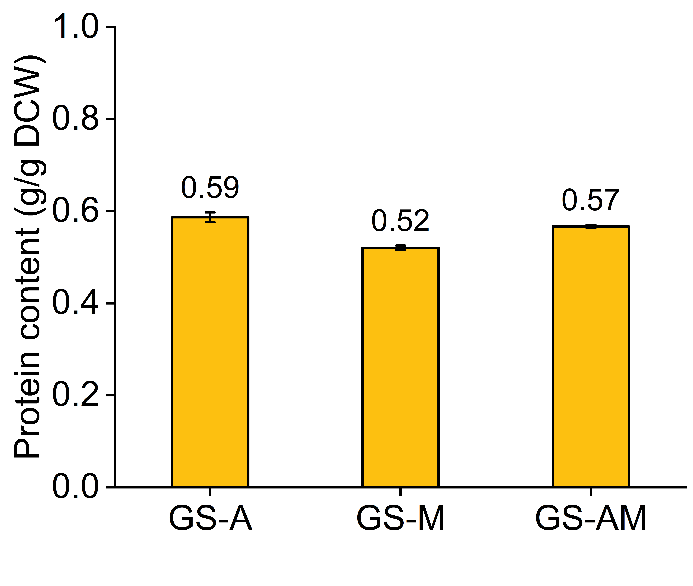


**Figure S1** **Crude protein content of the biomass from the erythritol-producing (GS-A), β-mannanase-producing (GS-M) and co-production (GS-AM) strain.** The crude protein content of the single-cell protein (SCP) biomass was determined by measuring total nitrogen. Cells were harvested by centrifugation, washed, and dried to a constant weight to obtain the dry biomass. Total nitrogen was quantified by the Kjeldahl method, and the crude protein content was calculated using a nitrogen-to-protein conversion factor of 6.25. Three independent biological replicates were performed, and results are expressed as grams of crude protein per gram of dry biomass (g/g).


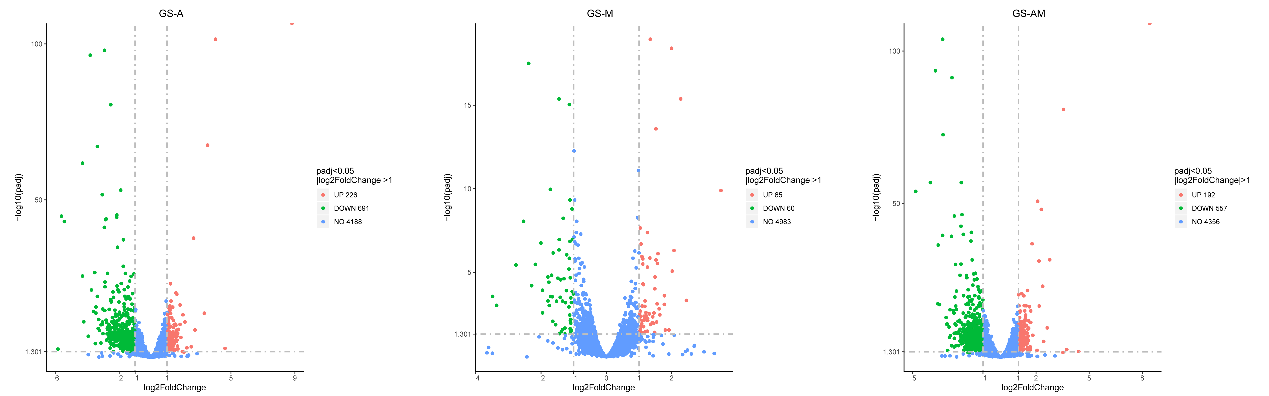


**Figure S2** **Volcano plots of differentially expressed genes in GS-A, GS-M and GS-AM strain.**


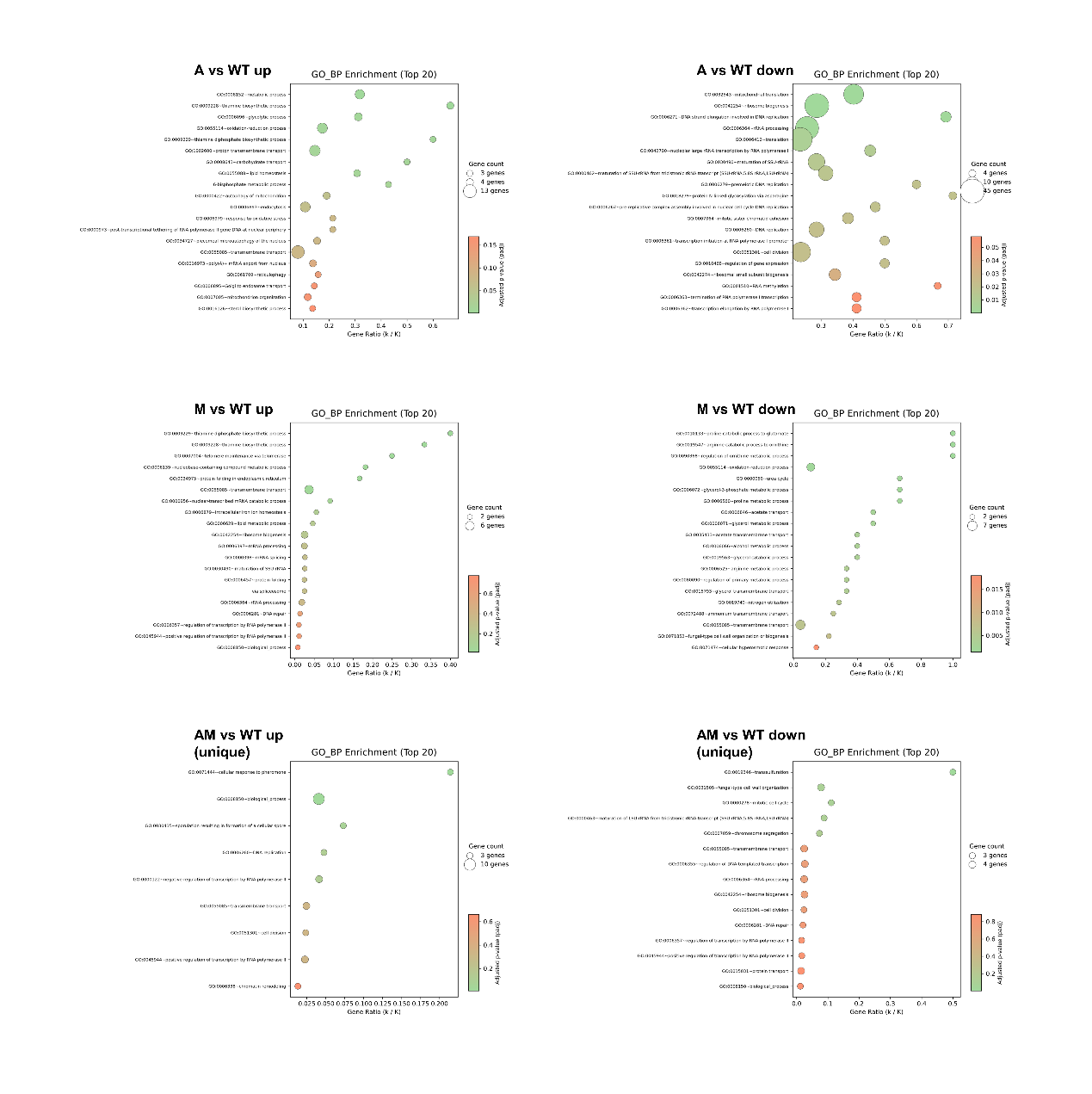


**Figure S3** **GO enrichment analysis of differentially expressed genes in GS-A, GS-M and GS-AM strain.**


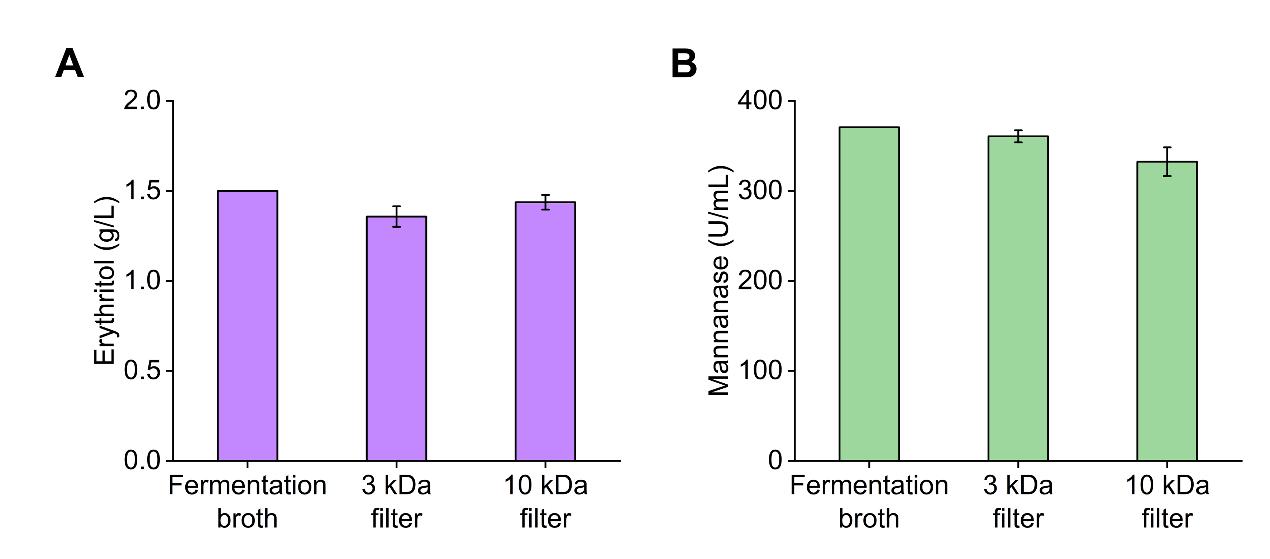


**Figure S4** **Erythritol concentration and β-mannanase activity before and after ultrafiltration.**


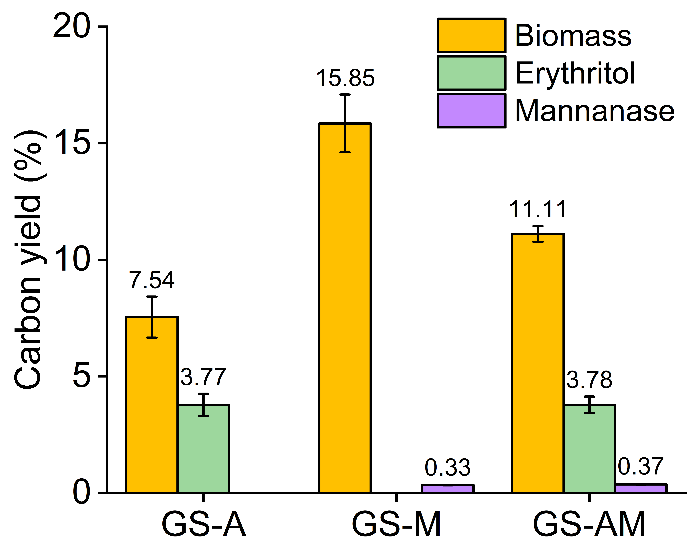


**Figure S5 Feedstock carbon balance of the single-production (GS-A, GS-M) and co-production (GS-AM) strain.** For each product, carbon yield was calculated as (product yield on methanol)×(product carbon mass fraction), expressed as a percentage of the carbon supplied per gram of methanol; carbon mass fractions were 0.39 (erythritol), 0.53 (β-mannanase) and 0.48 (biomass), with methanol taken as 0.375. β-mannanase mass was estimated from activity (6336 U/mL equivalent to 2.2 g/L protein; Zhu et al., 2011). The bars represent only product-associated carbon; the remaining methanol carbon (about 84%–89%) corresponds mainly to CO_2_ and is not shown.


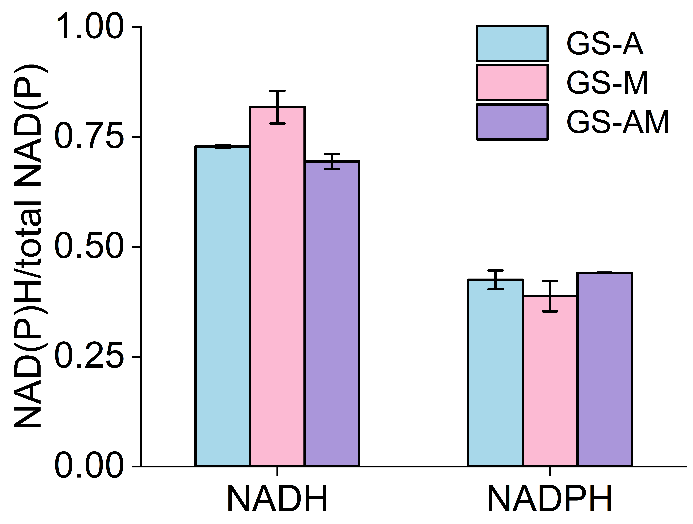


**Figure S6 Determination of intracellular NAD(P)H levels in GS-A, GS-M and GS-AM strain.**

**Table S1 Preliminary feedstock-based estimation of the product value per kilogram methanol for the tri-valorization strain (GS-AM), β-mannanase-producing strain (GS-M) and erythritol-producing strain (GS-A).**

| **Strain** | **Product** | **Yield**  **(U or g product per g MeOH)** | **Unit price**  **(USD per U or g product)** | **Value**  **(USD per kg MeOH)** | **Total value**  **(USD per kg MeOH)** | **Methanol cost**  **(USD per kg MeOH)** | **Value/cost ratio** |
| --- | --- | --- | --- | --- | --- | --- | --- |
| **GS-AM** | Mannanase | 7523.23 | 7.40×10^−8^ | 0.5565 | **0.6949** | 0.286 | **2.43×** |
|  | Erythritol | 0.03634 | 2.22×10^−3^ | 0.0806 |  |  |  |
|  | SCP | 0.08683 | 6.66×10^−4^ | 0.0578 |  |  |  |
| **GS-A** | Erythritol | 0.03627 | 2.22×10^−3^ | 0.0805 | **0.1197** | 0.286 | **0.42×** |
|  | SCP | 0.05890 | 6.66×10^−4^ | 0.0392 |  |  |  |
| **GS-M** | Mannanase | 6789.73 | 7.40×10^−8^ | 0.5022 | **0.5846** | 0.286 | **2.04×** |
|  | SCP | 0.12383 | 6.66×10^−4^ | 0.0824 |  |  |  |

Notes: Product titers were taken from the 1-L fed-batch fermentations reported in this study. Unit prices (USD): β-mannanase, 7.40×10^−8^ USD per enzyme activity unit (U); erythritol, 2.22×10^−3^ USD g^−1^; SCP, 6.66×10^−4^ USD g^−1^. Methanol cost was taken as 0.286 USD per kg, representing a representative reference price for methanol (China spot market, as of mid-2025). Value/cost ratio equals total product value (USD per kg methanol) divided by the methanol cost; a ratio >1 indicates that the feedstock-based product value exceeds the methanol cost. Only the raw-material cost of methanol is considered; utilities, labour, equipment depreciation and downstream separation are not included in this estimation.

**Reference**

Zhu T, You L, Gong F, Xie M, Xue Y, Li Y, Ma Y (2011) Combinatorial strategy of sorbitol feeding and low-temperature induction leads to high-level production of alkaline β-mannanase in Pichia pastoris. Enzyme Microb Technol 49:407-412.
